# Method Optimization: Analysis of Benzbromarone and Tolfenamic Acid in Citrus Tissues and Soil Using Liquid Chromatography Coupled With Triple-Quadrupole Mass Spectrometry

**DOI:** 10.3389/fpls.2020.00222

**Published:** 2020-03-06

**Authors:** Dan Zhang, Danilo R. da Silva, Timothy J. Garrett, Claudio F. Gonzalez, Graciela L. Lorca

**Affiliations:** ^1^Department of Microbiology and Cell Science, Genetics Institute, Institute of Food and Agricultural Sciences, University of Florida, Gainesville, FL, United States; ^2^Department of Pathology, Immunology and Laboratory Medicine, University of Florida, Gainesville, FL, United States

**Keywords:** benzbromarone, tolfenamic acid, agricultural products, citrus, liquid-liquid extraction, LC-MS/MS

## Abstract

Herein, an analytical method was developed for extraction and quantification of benzbromarone and tolfenamic acid in citrus and soil matrices using liquid-liquid extraction followed by liquid chromatography-triple quadrupole-tandem mass spectrometry analysis. The compounds were extracted using 0.1% formic acid in 6:4 ethyl acetate and n-hexane solution, and the analytes were separated using a mixture of 0.1% formic acid in ultrapure water and 0.1% formic acid in acetonitrile as mobile phase. A six-point in-matrix calibration curve was constructed providing good linearity with coefficients of determination *R*^2^ ≥ 0.98. The limits of detection and quantification for benzbromarone and tolfenamic acid were 3.0 and 10.0 μg/kg in roots, peel, juice, and soil, and 4.0 and 12.0 μg/kg for leaves samples, respectively. The method yielded excellent recoveries between 81.3 and 101.2%, with relative standard deviation ≤9.5% in the matrices. The developed technique provides a simple and sensitive method for the determination of the chemicals and can be applied to agricultural practices.

## Introduction

Huanglongbing (HLB), or citrus greening disease, is one of the most devastating citrus diseases affecting the world citrus production today (Bové, 2006). The disease originated in China and was first reported in the United States in 2005. Many other countries, such as Brazil, India, Iran, Cuba, the Dominican Republic, and Ethiopia have subsequently reported HLB infection (Tian et al., 2014). HLB is a highly contagious disease caused by the fastidious, phloem-limited bacteria *Liberibacter asiaticus* and transmitted by a psyllid vector (*Diaphorina citri*) (Jagoueix et al., 1994). After infection, the susceptible tree shows symptoms of the disease such as stunted growth, bitter and irregular sized fruit, premature fruit drop and blotchy mottle on the leaves. Over time, the infection progresses to tree death. Due to these symptoms, HLB has caused loss in revenues from reduced tree productivity. Although accurate values of production loss are difficult to calculate due to fluctuations in prices as well as yields, from 2000 to 2017, fresh and processed fruit utilization decreased by 71 percent (Court et al., 2018).

The lack of an axenic culture of *L. asiaticus* has hindered the identification and/or development of antimicrobial compounds that could effectively treat the infection. Using bioinformatics coupled with biochemical assays, we were able to identify two compounds, benzbromarone and tolfenamic acid, that could be used as a antimicrobial agents against *L. asiaticus* (Pagliai et al., 2014; Gardner et al., 2016). We found that, in *L. asiaticus*, benzbromarone acts by inhibiting the activity of the transcription factor LdtR, while tolfenamic acid modulates the activity of the transcription accessory protein PrbP. We found that the spray application of these compounds in citrus shoots and small citrus trees led to a significant reduction in the transcriptional activity of *L. asiaticus* (Pagliai et al., 2014; Gardner et al., 2016).

Benzbromarone (3,5-dibromo-4-hydroxyphenyl)-[(2-ethyl-1-benzofuran-3-yl)methanone] and tolfenamic acid (2-[(3-chloro-2-methylphenyl)amino]benzoic acid) are currently approved for their use in animal and/or human treatments. Benzbromarone is registered in over 20 countries throughout Asia, South America, and Europe and is used for the treatment of gout (Heel et al., 1977; Lee et al., 2008). Tolfenamic acid is a non-steroidal anti-inflammatory drug (NSAID) widely used in veterinary medicine to treat inflammation, pain, and fever (Smith et al., 2008). However, their use in agricultural practices has not yet been approved.

While the repurposing of these pharmaceutical drugs into new antimicrobials is very attractive, accurate methods that allow their determination in plant and soil substrates is needed to comply with good agricultural practices (GAP) and with domestic and international safety requirements. The potential application of benzbromarone and tolfenamic acid in citrus groves may lead to accumulation of these compounds in citrus products that may feed into human and animal consumption. Concurrently, the environmental impact due to the spray of the chemicals in this agricultural practice leaching into the soil and roots could become another source of contamination not only to humans but also to wildlife. Therefore, it is necessary to develop a reliable and sensitive method to extract and quantify both benzbromarone and tolfenamic acid in citrus leaves, fruit peel, juice, roots, and soil prior to its widespread use as a treatment for HLB.

Detection methods for benzbromarone and tolfenamic acid have been developed for several animal and human tissues as well as wastewater (Beyer et al., 2005; Gallo et al., 2008; Guan et al., 2016) however, methods for the simultaneous analysis of benzbromarone and tolfenamic acid from plant tissue are not available.

In this study, we describe a Liquid-Liquid Extraction (LLE) method for the simultaneous extraction of both compounds, benzbromarone and tolfenamic acid, and detection with the coupling of liquid chromatography with triple quadruple tandem mass spectrometry (LC-MS/MS) resulting in high specificity and sensitivity detection in citrus plant tissues and soil samples.

## Materials and Methods

### Chemicals and Reagents

Benzbromarone and tolfenamic acid analytical standards were purchased from Sigma (St. Louis, MO, United States). HPLC grade ethyl acetate, *n*-hexane, 0.1% formic acid in water, and 0.1% formic acid in acetonitrile were prepared from chemicals provided by Fisher Scientific (Pittsburgh, PA, United States). 0.1% formic acid in ethyl acetate and *n*-hexane (6:4, *v/v*) was prepared by diluting 1 mL formic acid in a 1 L flask containing 600 mL ethyl acetate and 400 mL *n*-hexane.

### Preparation of Standard Stock Solution

Ten mg of benzbromarone or tolfenamic acid were separately prepared in volumetric flasks by dissolving the chemical in 10 mL methanol and acetonitrile (1:1, *v/v*), yielding 1000 μg/mL stock solution. The intermediate and working solutions were prepared by further dilution with the same solvent, yielding variable concentrations ranging from 10.0− 60.0 μg/kg for roots, fruit peel, juice, and soil, and 12.0− 72.0 μg/kg for leaves for constructing the calibration curves. All standard solutions were stored at 4°C for an extended period (>6 months) with no apparent degradation of the compounds observed (data not shown).

### Sample Preparation

The different matrices (citrus leaves, roots, fruit peels, juice, and soil) were collected from mature citrus trees from groves in South Florida and stored at −80°C immediately. 15–20 mature leaves were collected randomly from multiple locations in the tree canopy. Root and soil samples were collected from the area within a 1-meter radius from the trunk of the citrus tree, a core of 30 cm was obtained from the area, and 50 mL tubes were filled with the soil and root. Fruit peel and fruit juices were collected from fruit that were ready to harvest by the time of sample collection. Citrus leaves, roots, fruit peels, and soil were lyophilized by a bulk tray dryer (Labconco, Kansas, MO, United States), then homogenized into a powder using a Geno/Grinder 2000 (Metuchen, NJ, United States). 300 mg of the resulting powder was weighed on a Mettler Toledo XS203S scientific scale (Bohemia, NY, United States) into a 15 mL centrifuge tube. For the optimization of the extraction method, samples were spiked with 30 μL of the standard solutions (1 μg/mL) and incubated at room temperature for 1 h. Non-spiked samples were used as background controls. Then, 3 mL 0.1% formic acid in ethyl acetate and *n*-hexane (6:4, *v/v*) were added, followed by vortexing (Fisher Dig Multi-Tube Vortexer, Pittsburgh, PA, United States) for 5 min. The mixture was centrifuged at 17,000 × *g* (Avanti J-26 XP, Beckman Coulter, United States) for 15 min at 4°C. The supernatant was decanted and transferred to a new 15 mL centrifuge tube and then dried completely under nitrogen at 40°C (Organomation, Berlin, MA, United States). Finally, the dried extract was reconstituted with 1 mL of ultra-pure water and acetonitrile (1:1, *v*/*v*). The mixture was vortexed for 1 min and centrifuged at 17000 × *g* for 15 min at 4°C and then filtered through a 0.2 μm syringe filter (Fisher Scientific, Pittsburgh, PA, United States). A volume of 5 μL was injected for LC–MS/MS analysis.

### LC-MS/MS Parameters

The analytes were identified using TSQ Quantum Access MAX Triple Quadrupole system coupled with ACCELA 1250 UHPLC system (Waltham, MA, United States). Analyses were carried out using heated-electrospray ionization in negative mode (HESI^–^) with selected reaction monitoring (SRM). The parameters for detection of benzbromarone and tolfenamic acid were vaporizer temperature, spray voltage, sheath gas pressure, aux gas pressure, capillary temperature, and collision energy.

Gradient separation was carried out using a Waters Symmetry (Milford, MA, United States) C18 (150 × 2.1 mm, 3.5 μm) column. The mobile phase consisted of 0.1% formic acid in water (A) and 0.1% formic acid in acetonitrile (B). All the operating conditions are shown in Table 2.

### Analytical Validation

Analytical parameters, including limits of detection (LOD) and quantification (LOQ), linearity, specificity, accuracy, and precision were determined to validate the method. LOD and LOQ were calculated as signal-to-noise (S/N) ratios of 3:1 and 10:1, respectively. Linearity was obtained by analyzing the standard solutions both in solvent and matrix at six-points over the range of 10.0 – 70.0 μg/kg. Specificity means the ability of a method to distinguish between the target analyte being measured in a blank sample from other interfering compounds at or around the retention time of the target analytes. Accuracy (expressed as recovery) was evaluated through spiking blank samples at three different concentrations, whereas precision was expressed as relative standard deviation (RSD ≤ 9.5%).

## Results

### Optimization of the Standard Solution Preparation

Methanol, acetonitrile, and a combination of methanol and acetonitrile (1:1, *v/v*) were tested for the standard solution preparation. Since a mixture of methanol and acetonitrile (1;1, *v/v*) had a higher detection intensity (benzbromarone peak area: 176,926 ± 0.1, tolfenamic acid: 40,448 ± 0.2) than methanol (149,546 ± 0.1, 37,803 ± 0.1) or acetonitrile alone (146,237 ± 0.1,38,576 ± 0.3) at 500.0 μg/kg, it was subsequently used for the preparation of standard solutions in this study.

### Optimization of Sample Preparation and Extraction

Leaves were spiked with both analytes at a concentration of 500.0 μg/kg and then incubated at room temperature for 1, 2, 4, and 8 h. There were no statistical differences between the 4 time-points. The recovery rates ranged between 89.1 – 106.6% (SD ≤ 8.2%). Because of the negligible differences, the 1 h incubation was chosen for equilibrating the samples.

Non-polar organic solvents are widely used for extracting the residues from food and agricultural samples (Lee et al., 2017). To evaluate the extraction efficiency for both analytes, leaf powder was extracted with 80% methanol, methanol, acetonitrile, methanol and acetonitrile (1:1, *v/v*), ethyl acetate and methanol (1:1, *v/v*), ethyl acetate and acetonitrile (1:1, *v/v*), ethyl acetate and *n*-hexane (1:1, *v/v*), and ethyl acetate and *n-*hexane (6:4, *v/v*). The best recovery was obtained in ethyl acetate and *n*-hexane (6:4, *v/v*) (85.5 ± 2.0 and 90.5 ± 4.3 percent for benzbromarone and tolfenamic acid, respectively) (Table 1). Further optimization was performed by testing the addition of 0.1% acetic acid, 0.1% formic acid, 0.1% phosphoric acid, and 10 mM ammonium acetate to ethyl acetate and *n*-hexane (6:4, *v/v*). It was found that 0.1% formic acid in ethyl acetate and *n*-hexane (6:4, *v/v*) yielded the best extraction efficiency (Table 1) and therefore was employed as the extraction solvent for further experiments.

**TABLE 1:** Optimization of the extraction efficiency for benzbromarone and tolfenamic acid in citrus matrices.

	**Recovery (%)**	
**Solvent**	**Benzbromarone**	**Tolfenamic acid**
80% methanol	0	0
Methanol	64.1 ± 5.0	74.4 ± 3.9
Acetonitrile	69.7 ± 0.6	76.0 ± 3.2
Methanol and acetonitrile (1:1)	70.2 ± 1.1	80.7 ± 3.1
Ethyl acetate and methanol (1:1)	82.1 ± 2.9	80.1 ± 3.2
Ethyl acetate and acetonitrile (1:1)	81.0 ± 1.5	83.5 ± 2.9
Ethyl acetate and hexane (1:1)	82.0 ± 2.4	86.6 ± 2.6
Ethyl acetate and hexane (6:4)	85.5 ± 2.0	90.5 ± 4.3
Ethyl acetate and hexane (6:4) 0.1% acetic acid	89.3 ± 0.9	91.2 ± 1.0
Ethyl acetate and hexane (6:4) 0.1% formic acid	94.7 ± 2.8	96.5 ± 1.3
Ethyl acetate and hexane (6:4) 0.1% phosphoric acid	91.4 ± 0.8	93.2 ± 1.8
Ethyl acetate and hexane (6:4) 10 mM ammonium acetate	89.9 ± 1.5	89.9 ± 0.2

### Optimization of LC-MS/MS Analysis

**FIGURE 1:**
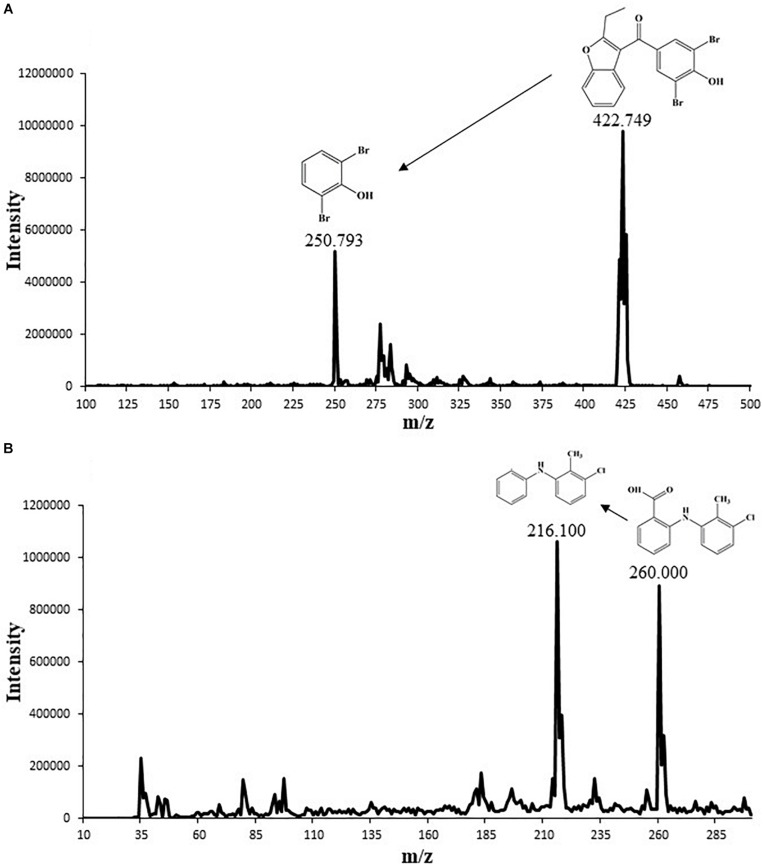
Mass spectrum chromatogram showing the molecular size of **(A)** benzbromarone (peak 422.749) and **(B)** tolfenamic acid (peak 260.000) along with their chemical structure and potential degradation fragment.

Both positive and negative heated-electrospray ionization (HESI^±^) modes were tested for quantification of benzbromarone and tolfenamic acid. Both analytes were detectable in negative ion mode; hence HESI^–^ mode was selected for simultaneous quantification. The MS/MS interface parameters and limits of the most sensitive transitions (422.7 > 250.8, 260.0 > 216.1 for tolfenamic acid and benzbromarone, respectively) were used for quantification and confirmation in SRM (Table 2). Based on these operating conditions, the structural analysis of benzbromarone and tolfenamic acid were performed (Figure 1).

**TABLE 2:** LC-MS/MS operating conditions.

**Parameter**	**Settings**			
**LC parameters**				
Mobile phase		A: 0.1% formic acid in water		
		B: 0.1% formic acid in acetonitrile		
		Time (min)	A (%)	B (%)
		0	80	20
		3	80	20
		5	20	80
		8	20	80
		10	80	20
		12	80	20
Flow rate		600 μL/min		
Column temperature		30°C		
Injection volume		5 μL		
Run time		12 min		
**MS-MS interface parameters**				
Ionization mode		Electrospray ionization (negative mode)		
Ion Source		HESI II		
Vaporizer temperature Spray voltage		350°C 3000 V		
Sheath gas pressure		50 AU		
Aux gas pressure		10 AU		
Capillary temperature		320°C		
**SRM parameters**				
Collision Pressure		1.5 mTorr (Argon)		
	MW	Monitored Reactions Precursor *m/z* > product *m/z*	t_*R*_ (min)	Collision Energy (V)
Benzbromarone	424.1	422.7 > 250.8	6.22	37
Tolfenamic acid	261.7	260.0 > 216.1	6.01	18

*t_*R*_, retention time.*

The column as well as the mobile phase were tested for the best separation and signal sensitivity of the chromatograms. Based on previous research, the separation was performed on a C18 separation column. Using this column, sharp and distinct chromatograms for both benzbromarone and tolfenamic acid were obtained. For the selection of the mobile phase, acetonitrile, methanol, and acetonitrile and methanol (1:1, *v/v*) were tested. Although clear separation was observed (data not shown) when methanol was used as the organic phase, higher intensity chromatograms were obtained when acetonitrile was used when compared to acetonitrile and methanol (1:1, *v/v*) or methanol alone. Then, 0.1% acetic acid, 0.1% formic acid, 0.5% formic acid, 1% formic acid, 0.1% phosphoric acid, and 10 mM ammonium acetate were tested as the aqueous phase. The best results were obtained using 0.1% formic acid in water (mobile phase A) and 0.1% formic acid in acetonitrile (mobile phase B); these were therefore selected for further analyses.

### Method Validation

#### Specificity

The specificity of the method was tested by analyzing control citrus leaves, roots, fruit peel, juice, and soil samples. As shown in the chromatograms, there were no apparent interference peaks at or around the retention time for the control (non-spiked samples) and spiked samples when compared to that of the standards (Supplementary Figures S1, S2).

#### Matrix Effect and Linearity

Matrix effect (ME) refers to a difference in mass spectrometric response for the tested analytes in standard solution vs. the response for the same analytes in matrix. Negative values represent the degree of suppression (%), whereas positive values represent the degree of enhancement (%). MEs estimated from two concentrations (500.0 and 1000.0 μg/kg) (*n* = 3) were ranged between −5.4% to 10.8% in all matrices; these MEs were very mild and negligible. Therefore, for accuracy, matrix-matched calibrations were used for quantification. Six concentration levels equivalent to 1, 2, 3, 4, 5, and 6-fold the LOQ (*n* = 4) were constructed to establish the calibration curves. For citrus roots, fruit peel, juice, and soil, the concentrations were 10.0, 20.0, 30.0, 40.0, 50.0, and 60.0 μg/kg for both benzbromarone and tolfenamic acid. Similarly, for citrus leaves, the concentrations were 12.0, 24.0, 36.0, 48.0, 60.0, and 72.0 μg/kg. Good linearity was achieved for each matrix-matched calibration, with a coefficient of determination (*R*^2^) ≥ 0.98 (Table 3).

**TABLE 3:** Performance parameters of benzbromarone and tolfenamic acid in different matrices.

**Analytes**	**matrix**	**Spiking level (μg/kg)**	**Precision**				**Calibration curve (μg/kg)**	***R*^2^**	**Linear range (μg/kg)**	**LOD (μg/kg)**	**LOQ (μg/kg)**
			**Intra-day (*n* = 3)**		**Inter-day (*n* = 9)**						
			**Recovery (%)**	**RSD (%)**	**Recovery (%)**	**RSD (%)**					
Benzbromarone	Leaves	12.0	87.7 ± 6.9	7.8	84.5 ± 4.4	5.2	*y* = 261727000x + 115820	0.991	12.0–72.0	4.0	12.0
		24.0	98.4 ± 0.4	0.4	97.1 ± 1.2	1.2					
	Root	10.0	93.9 ± 3.8	4.0	93.6 ± 2.4	2.5	*y* = 306129000x ± 1206000	0.99	10.0–60.0	3.0	10.0
		20.0	99.5 ± 0.4	0.4	90.5 ± 2.6	2.9					
	Peel	10.0	88.2 ± 8.3	9.5	90.6 ± 1.3	1.4	*y* = 270863000x + 1816500	0.996			
		20.0	101.2 ± 0.5	0.5	92.4 ± 1.8	2.0					
	Juice	10.0	92.6 ± 3.0	3.2	91.2 ± 1.5	1.6	*y* = 231101000x + 2163200	0.997			
		20.0	94.5 ± 5.3	5.6	95.0 ± 3.8	4.0					
	Soil	10.0	90.3 ± 3.5	3.8	88.6 ± 1.7	2.0	*y* = 200148000x + 2496500	0.985			
		20.0	100.8 ± 6.1	6.1	94.7 ± 3.4	3.6					
Tolfenamic acid	Leaves	12.0	100.6 ± 3.0	3	81.3 ± 1.3	1.5	*y* = 1000000x – 6649.6	0.988	12.0–72.0	4.0	12.0
		24.0	92.8 ± 4.8	5.1	95.3 ± 0.3	0.3					
	Root	10.0	92.0 ± 1.1	1.2	98.5 ± 2.0	2.1	*y* = 1000000x – 5783.2	0.985	10.0–60.0	3.0	10.0
		20.0	97.1 ± 2.9	3.0	95.0 ± 0.4	0.4					
	Peel	10.0	92.0 ± 1.1	1.2	98.5 ± 2.0	2.1	*y* = 1000000x – 3932.5	0.981			
		20.0	97.1 ± 2.1	2.3	95.0 ± 0.4	0.4					
	Juice	10.0	96.2 ± 1.4	1.4	93.7 ± 3.4	3.6	*y* = 1000000x – 6999.2	0.98			
		20.0	94.5 ± 2.2	2.2	92.7 ± 7.6	8.2					
	Soil	10.0	86.1 ± 4.6	5.4	82.2 ± 2.3	2.8	*y* = 1000000x – 5553.4	0.983			
		20.0	90.6 ± 2.2	2.4	92.2 ± 7.3	7.9					
											

#### Accuracy and Precision

The accuracy and precision were measured as recovery and RSD, respectively. Samples were spiked at 10.0 and 20.0 μg/kg in citrus roots, leaves, fruit peel, juice, and soil samples for both benzbromarone and tolfenamic acid. Excellent recovery was obtained for benzbromarone (between 84.5% ± 4.4% ∼ 100.8% ± 6.1% with RSD ≤ 9.5%) and for tolfenamic acid (81.3% ± 1.3% ∼ 100.6% ± 3.0% with RSD ≤ 8.2%) for both inter- and intra-day analysis in the different matrices, respectively (Table 3).

#### LOD and LOQ

The limits of detection (LOD) and limits of quantification (LOQ) were calculated as 3 and 10 times the signal-to-noise ratio, respectively. The LOD was 3.0 μg/kg, whereas the LOQ was 10.0 μg/kg, for both benzbromarone and tolfenamic acid in roots, peel, juice, and soil (Table 3). A slightly higher LOD and LOQ of 4.0 and 12 μg/kg, respectively, was observed in leaf samples for both compounds. This increase may be due to the presence of a residual green pigmentation when the extraction method developed was used on the leaf samples, possibly affecting the background of the chromatograms. Since the detection limits were in the μg/kg range, we can conclude that the analytical method has a high sensitivity to detect and quantify the residues in environmental samples.

### Method Application

**FIGURE 2:**
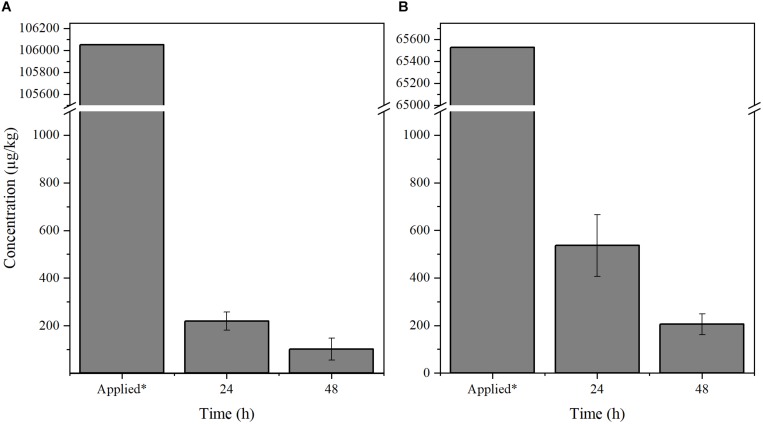
Quantification of **(A)** benzbromarone and **(B)** tolfenamic acid from citrus tissue. Leaf samples were collected from 2-year-old *Citrus sinensis* trees (*n* = 3) sprayed with benzbromarone and tolfenamic acid, respectively. The concentration of both compounds were determined using the described LC-MS/MS method 24 and 48 h post-spray. *Concentration of the chemicals applied to saturation.

We next tested if the chemicals may be efficiently detected in small citrus plants. Two-year-old healthy *Citrus sinensis* trees planted on an experimental plot located at the University of Florida were used for the experiment. The trees were exposed to environmental conditions such as direct sunlight, weather, and wind. Benzbromarone and tolfenamic acid were delivered by spray to the trees until saturated (*n* = 3). Leaf samples (5–10 leaves) were then collected, and the concentration of each chemical was evaluated 24 and 48 h post-spray (Figure 2). Previous phytotoxicity assays with tolfenamic acid and benzbromarone have shown no adverse effect when citrus plants are sprayed at 65,526 μg/kg and 106,052 μg/kg (250 μM), respectively (Pagliai et al., 2014; Gardner et al., 2016). Tolfenamic acid was detected at ∼536 μg/kg after 24 h post-spray, while it decreased to 138 μg/kg after 48 h post-spray (Figure 2B). A similar effect was observed with benzbromarone when the trees were sprayed to saturation (Figure 2A). These observations confirm that the method developed can be used to detect both chemicals from citrus plant tissue even when exposed to environmental conditions.

## Discussion

In this study, we described the optimization of an extraction and analytical method for the simultaneous detection and quantification of benzbromarone and tolfenamic acid in citrus plant and soil matrices. Since these compounds are proposed for the management of HLB, the detection and evaluation of their concentration is critical.

Previous studies have reported the quantification of benzbromarone in rats and human tissues using gas chromatography-mass spectrometry (GC-MS) and high-performance liquid chromatography-quadruple time of flight mass spectrometry (HPLC-Q-TOF), respectively (Beyer et al., 2005; Wu et al., 2012). Beyer et al. (2005) used GC-MS analysis methods for the detection of benzbromarone with an estimated value of 0.05 mg/L for LOD in human urine. The method we have developed is 10 times more sensitive with an LOD as low as 3.0 μg/kg (∼0.003 mg/L).

Tolfenamic acid has been detected in bovine milk samples by LC-MS/MS methods with LODs of 53.0−54.0 μg/kg (Malone et al., 2009; Jedziniak et al., 2012; van Pamel and Daeseleire, 2015), and LOQ of 4.0 μg/kg (Gallo et al., 2008). In bovine meat by LC-MS/MS methods, the LODs were 57.0 – 59.0 μg/kg (Van Hoof et al., 2004; van Pamel and Daeseleire, 2015). Tolfenamic acid was also detected in bovine milk by HPLC methods with a LOQ of 10.0 μg/kg (Gallo et al., 2010). The method described in this study allows for higher sensitivity (3.0 μg/kg) compared to previous studies for the individual detection of tolfenamic acid.

While higher sensitivity has been achieved for the detection of tolfenamic acid using a UPLC-MS/MS method with of LOD 0.076 ng/mL and LOQ 0.216 ng/mL in wastewater (Guan et al., 2016), the method required concentration of the samples using solid-phase extraction (SPE) methods. SPE has been reported for the extraction and purification of benzbromarone and tolfenamic acid. A Vac-Master V-10 cartridge was used for extraction of benzbromarone in human urine resulting in an LOD of 0.05 mg/mL (Beyer et al., 2005). Similarly, C18 end-capped SPE cartridges, Oasis HLB SPE cartridges, Bond-Elut SAX SPE cartridges, and Sep-Oak NH_2_ SPE cartridges were used for extraction of tolfenamic acid from bovine milk, bovine muscle, and human urine, respectively, with LOQ as low as 4 μg/kg (Mikami et al., 2000; Van Hoof et al., 2004; Beyer et al., 2005; Gallo et al., 2008, 2010; Jedziniak et al., 2012). Although SPE can effectively eliminate interference of other substances found in the matrices and is commonly used in other studies, the SPE procedure incorporates several steps that lead to longer processing times compared to LLE and can be cost prohibitive.

Here we demonstrate a simple, sensitive, and accurate LC-MS/MS method for the determination of benzbromarone and tolfenamic acid for agricultural practices. The method developed was validated in citrus leaves, roots, fruit peel, juice, and soil. We found that a simple mixture of acidified ethyl acetate and *n*-hexane as an extraction solvent gave excellent recovery for the tested analytes. No further purification procedure was necessary, significantly decreasing the cost of the assay. The developed method was tested and validated by both spiking the matrices with the chemicals and in planta analysis. Therefore, the newly developed method can be used for routine simultaneous analysis of benzbromarone and tolfenamic acid in citrus plant matrices with the potential of expansion to other plant matrices.

In summary, our optimized method allows the simultaneous determination of benzbromarone and tolfenamic acid in plant derived matrices and soil. Moreover, the unit factor of μg/kg provides a low level for LOD and LOQ to ensure high sensitivity of detection across the different citrus tissues.

## Data Availability Statement

The datasets generated for this study are available on request to the corresponding author.

## Author Contributions

DZ performed the research. DZ and DS analyzed the data. TG and CG contributed to the discussion and reviewed the manuscript. DZ, DS, and GL wrote the manuscript. CG and GL conceived the study.

## Conflict of Interest

The authors declare that the research was conducted in the absence of any commercial or financial relationships that could be construed as a potential conflict of interest.

## Supplementary Material

FIGURE S1 Chromatograms showing the **(A)** blank, **(B)** benzbromarone standard (1 μg/mL) and **(C)** tolfenamic acid standard (1 μg/mL) utilizing the developed quantification method.

FIGURE S2 Chromatograms of **(A)** unspiked, **(B)** benzbromarone spiked (1 μg/mL), and **(C)** tolfenamic acid spiked (1 μg/mL) matrix, as indicated on the right side of the panel.
