# Supplementary figures and images for: Method Optimization: Analysis of Benzbromarone and Tolfenamic Acid in Citrus Tissues and Soil Using Liquid Chromatography Coupled With Triple-Quadrupole Mass Spectrometry
Source: Front Plant Sci. 2020 Mar 6;11:222. doi: 10.3389/fpls.2020.00222 (PMC7068813; doi:10.3389/fpls.2020.00222)

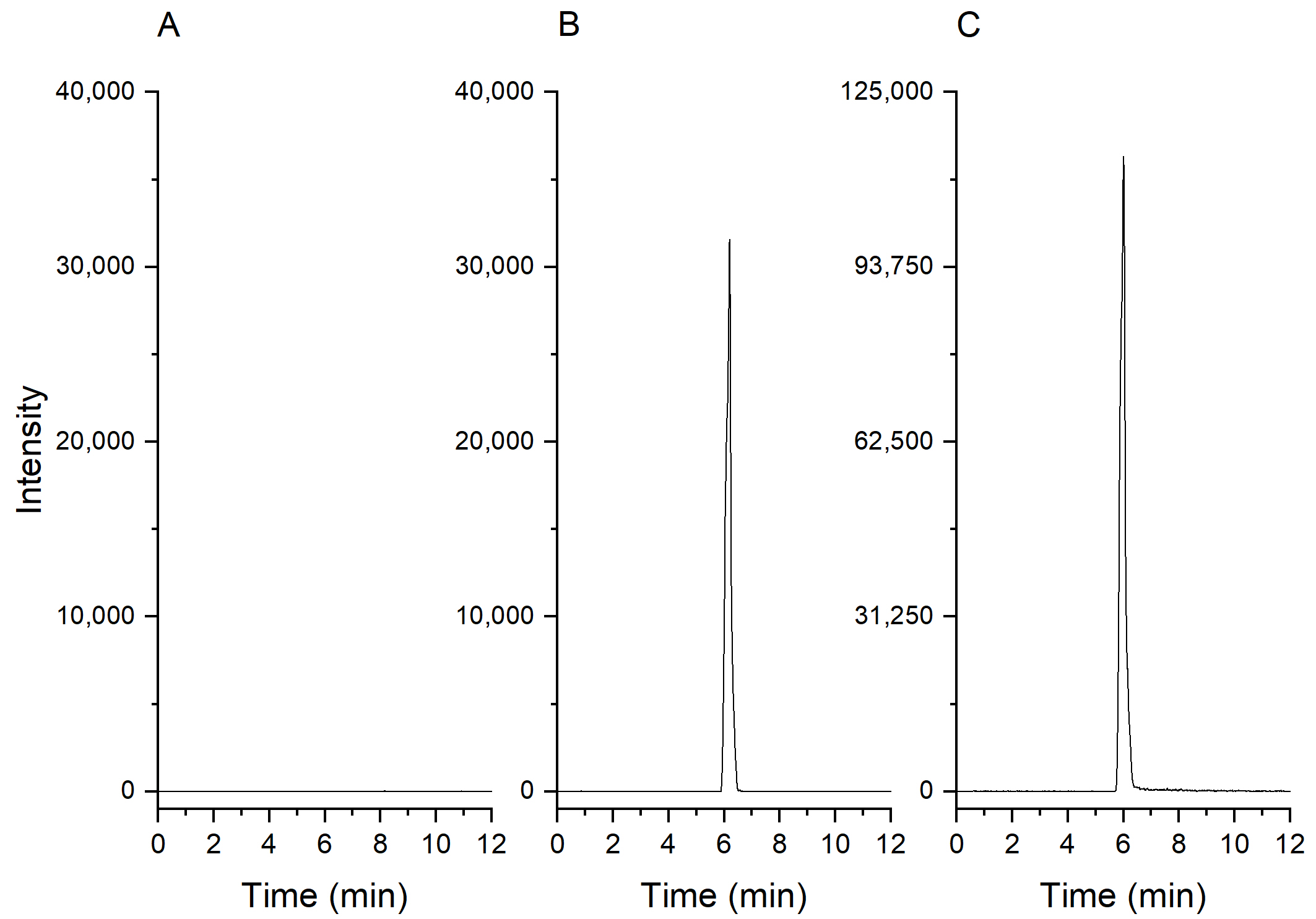

Supplement: FIGURE S1 — Chromatograms showing the (A) blank, (B) benzbromarone standard (1 μg/mL) and (C) tolfenamic acid standard (1 μg/mL) utilizing the developed quantification method. [file Image_1.JPEG]

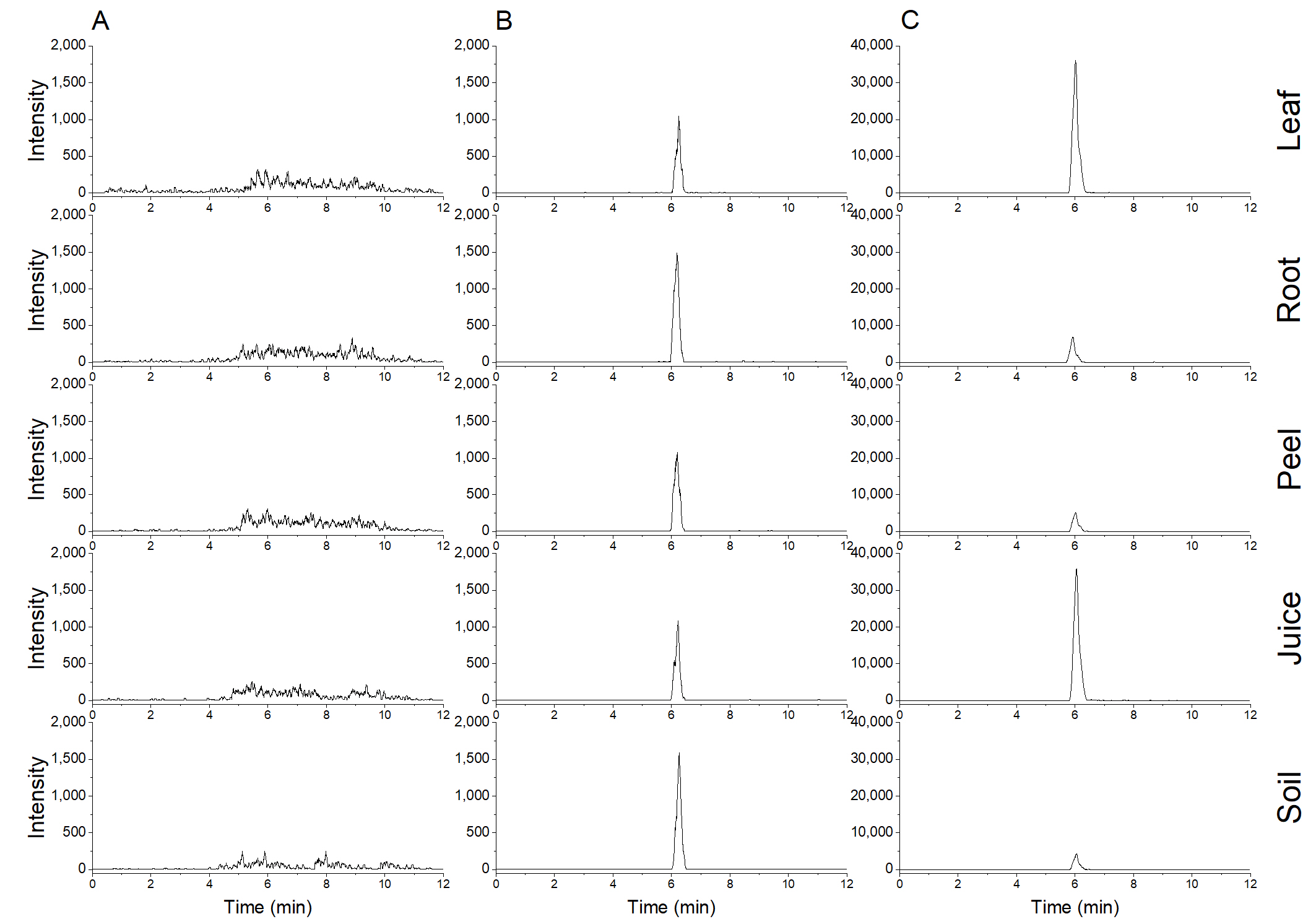

Supplement: FIGURE S2 — Chromatograms of (A) unspiked, (B) benzbromarone spiked (1 μg/mL), and (C) tolfenamic acid spiked (1 μg/mL) matrix, as indicated on the right side of the panel. [file Image_2.JPEG]
